# Supplementary material for: Reemergence of high-Tc superconductivity in the (Li1-xFex)OHFe1-ySe under high pressure
Source: Nat Commun. 2018 Jan 25;9:380. doi: 10.1038/s41467-018-02843-7 (PMC5785538; doi:10.1038/s41467-018-02843-7)
Supplement: Supplementary file 1 — Supplementary Information [file 41467_2018_2843_MOESM1_ESM.pdf]

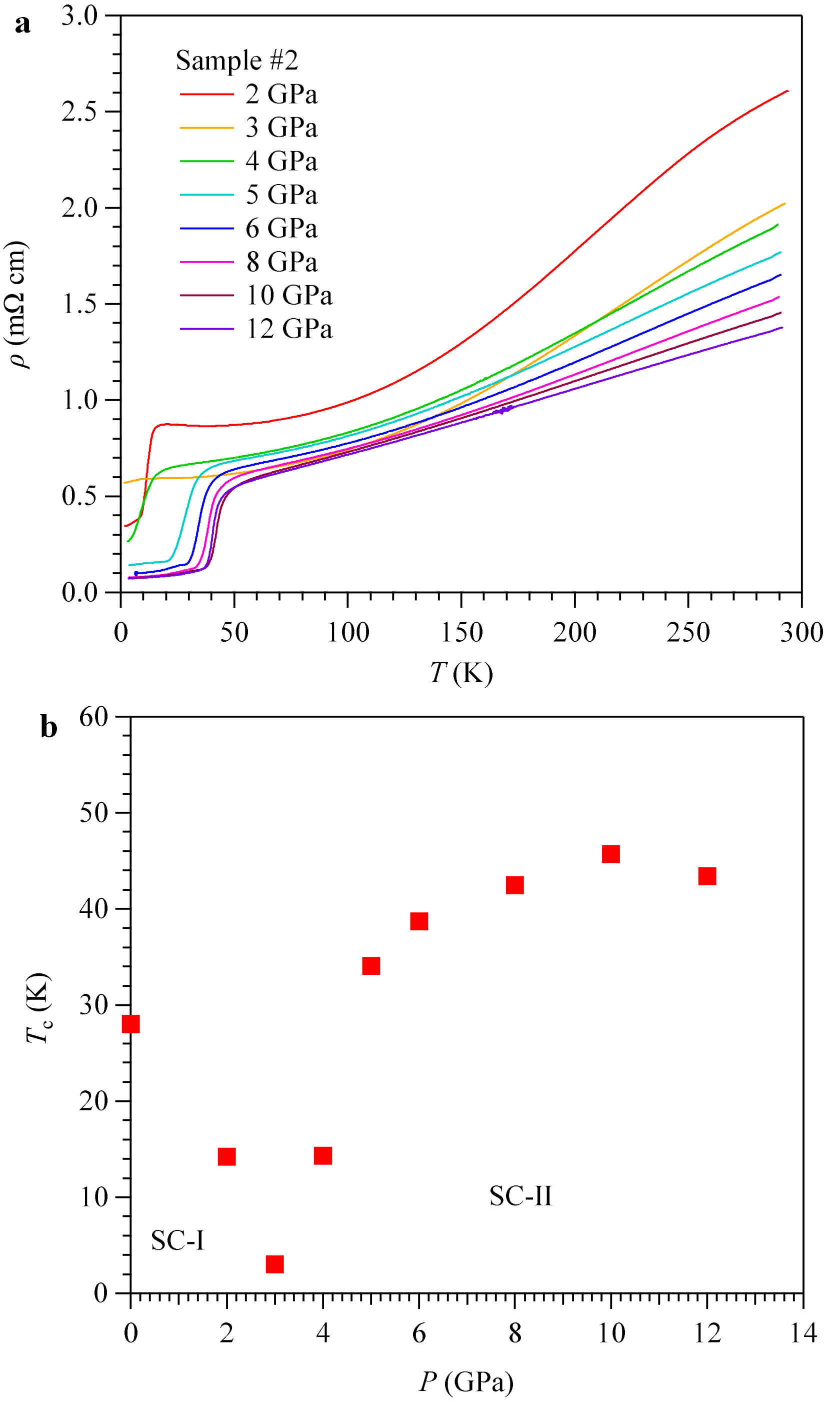

Supplementary Figure 1. High-pressure resistivity on a second $(Li_{1-x}Fe_x)OHFe_{1-y}Se$ sample with an ambient-pressure T_c = 28 K. (**a**) Temperature-dependence of resistivity $\rho(T)$ under different pressures for the Sample #2. (**b**) Pressure dependence of T_c for the Sample #2 evidencing the emergence of the second superconducting (SC-II) phase above 3 GPa.

Supplementary Note 1. Density functional calculations

We did first principles calculations as a function of pressure to investigate changes in the electronic structure and magnetic tendencies. The calculations were done primarily using the general potential linearized augmented planewave (LAPW) method [1] as implemented in the WIEN2k code [2] and the PBE generalized gradient approximation (PBE-GGA) [3]. We did calculations for stoichiometric LiOHFeSe using structures determined as a function of pressure from first principles. The structure optimization was done using the VASP code using projector augmented wave pseudopotentials [4], which has an efficient and accurate implementation of the stress tensor. These optimizations were done without magnetism. We also performed calculations for the hypothetical LiFFeSe, with the same structures as LiOHFeSe, replacing OH by F. While the chemistry of this system is different from LiOHFeSe, we verified that from an electronic structure point of view it is practically identical, confirming that the LiOH layers in the lattice play no active role other than charge balance and stabilization of the structure. For LiOHFeSe we used LAPW sphere radii of 1.7 bohr for Fe and Se, 1.3 bohr for Li and 0.9 bohr for O and H. These small radii were needed due to the short O-H bond length.

Electronic structure results are shown in Supplementary Figure 2. At ambient pressure, we find five sheets of Fermi surface, all of which are very two dimensional. These are two main hole cylinders and two electron cylinders, which are common to undoped Fe-based superconductors, and a small additional hole cylinder. Fermi surfaces at 0, 5, and 10 GPa are shown in Supplementary Figure 2(a). As seen the main difference under pressure is the disappearance of the small hole section. The variation of the Fermi surface pockets with pressure is shown in Supplementary Figure 2(b). The pressure range of enhanced superconductivity is the range where the small hole section becomes very small, and therefore where the hole and electron volumes compensate. The other clear trend under pressure is that the two main hole sections gradually become very similar to each other both in shape and area. There is no noticeable tendency for the electronic structure to become significantly three dimensional in the pressure range studied. The evolution of the density of states near the Fermi energy, $N(E_F)$, is shown in Supplementary Figure 2(c). The main effect of pressure is a small gradual decrease in $N(E_F)$.

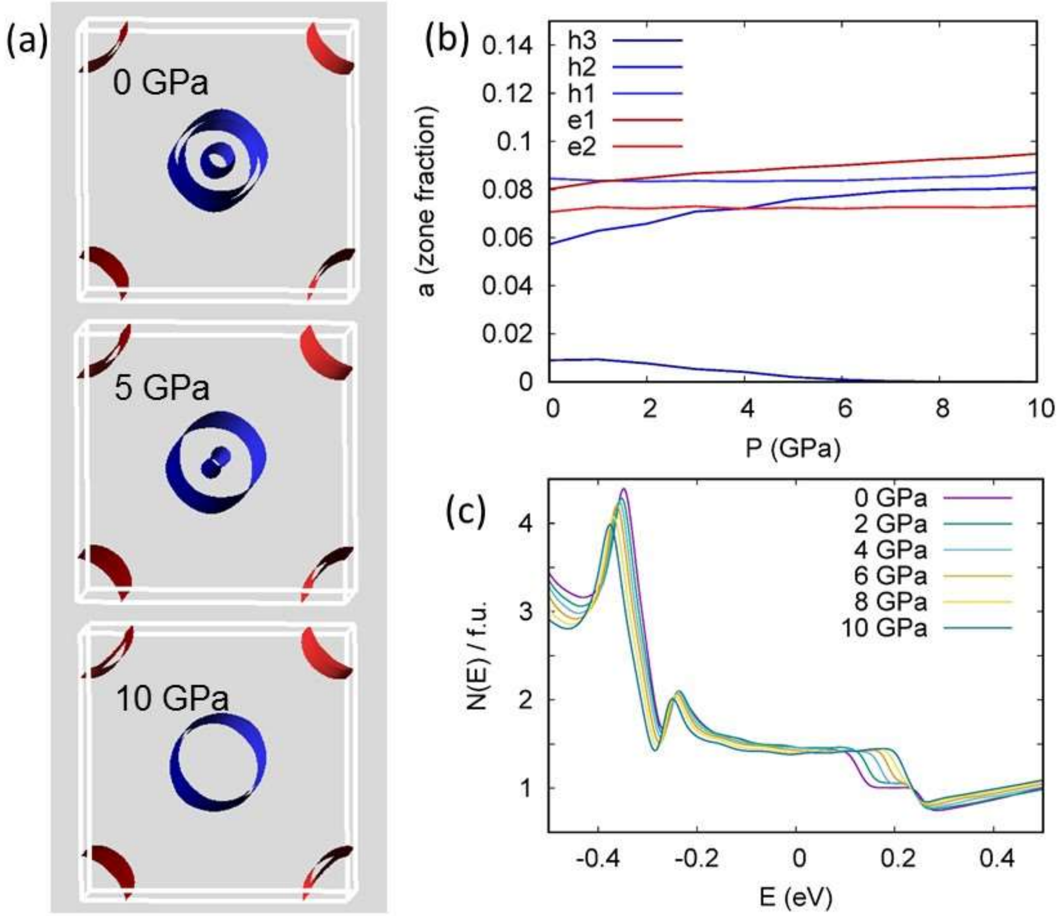

Supplementary Figure 2. Evolution of the electronic structure of LiOHFeSe with pressure based on density functional calculations. (**a**) Evolution of the Fermi surface at 0, 5, and 10 GPa. Hole sections are shown in blue and electron sections in red. (**b**) Fermi surface volumes, a, in fractions of the zone, for three hole sections, h1, h2, and h3, and for two electron sections, e1 and e2. (**c**) Total density of states per formula unit, $N(E)$/f.u., at various pressures.

Supplementary References

1. Singh, D. J. & Nordstrom, L. Planewaves, pseudopotentials and the LAPW method, Second Edition. (Springer, Berlin, 2006).

2. Blaha, P., Schwarz, K., Madsen, G., Kvasnicka, D. & Luitz, J. WIEN2k, An augmented planewave + local orbitals program for calculating crystal properties (TU Wien, Austria, 2001).

3. Perdew, J. P., Burke, K., & Ernzerhof, M. Generalized gradient approximation made simple. *Phys. Rev. Lett.* **77**, 3865 (1996).

4. Kresse, G. & Joubert, D. From ultrasoft pseudopotentials to the projector augmented-wave method. *Phys. Rev. B* **59**, 1758 (1999).
